# Time in Range, as a Novel Metric of Glycemic Control, Is Reversely Associated with Presence of Diabetic Cardiovascular Autonomic Neuropathy Independent of HbA1c in Chinese Type 2 Diabetes

**DOI:** 10.1155/2020/5817074

**Published:** 2020-02-06

**Authors:** Qingyu Guo, Pu Zang, Shaoying Xu, Wenjing Song, Zhen Zhang, Chunyan Liu, Zhanhong Guo, Jing Chen, Bin Lu, Ping Gu, Jiaqing Shao

**Affiliations:** ^1^Jinling Hosp Dept Endocrinology, Nanjing Univ, Sch Med, Nanjing, China; ^2^Jinling Hosp Dept Endocrinology, Southeast Univ, Sch Med, Nanjing, China; ^3^Shanghai Sixth People's Hospital East, Shanghai, China; ^4^The Seventh Affiliated Hospital, Sun Yat-sen University, Shenzhen, China; ^5^Affiliated Hospital of Jiangnan University, Wuxi, China; ^6^Jinling Hosp Dept Endocrinology, Nanjing Med Univ, Nanjing, China

## Abstract

**Objective:**

The objective of this study is to investigate the relationship between time in range (TIR), a new metric of continuous glucose monitoring (CGM) and cardiovascular autonomic neuropathy (CAN) in individuals with type 2 diabetes mellitus (T2DM).

**Methods:**

A total of 349 individuals with T2DM were enrolled in this study. Evaluating by the standard cardiac autonomic reflex tests (CARTs), there were 228 diabetic individuals without cardiovascular autonomic neuropathy (without confirmed CAN) including absent CAN (*n* = 83 cases) and early CAN (*n* = 83 cases) and early CAN (*n* = 83 cases) and early CAN (*n* = 83 cases) and early CAN (

**Results:**

The total presence of CAN was 34.67% (definite CAN 31.23% and severe CAN 3.44%). Patients with more severe CAN had lower TIR (*P* < 0.001). With increasing quartiles of TIR, the presence of CAN by severity declined (*P* < 0.001). With increasing quartiles of TIR, the presence of CAN by severity declined (*P* < 0.001). With increasing quartiles of TIR, the presence of CAN by severity declined (*P* < 0.001). With increasing quartiles of TIR, the presence of CAN by severity declined (

**Conclusion:**

TIR is associated with the presence of CAN independent of HbA1c and GV metrics in Chinese type 2 diabetes.

## 1. Introduction

With the rapidly developing technology and increasing use of continuous glucose monitoring (CGM), CGM has been the optimal method to get information on glycemic profile throughout the day. CGM with high accuracy can reflect an individual's glycemic status including hyperglycemia, hypoglycemia, glycemic variability (GV), and daily patterns of glycemia, which provides indication for the treatment of diabetes mellitus [1]. The capability to grade blood glucose levels into different ranges has been convenient likely due, in great part, to increasing use of CGM. The emerging metric time in range (TIR) typically refers to the time percent during a 24-hour period when the glucose is in the range of 3.9-10 mmol/L. A recent international consensus conference proposed that TIR should be the key CGM-derived metric describing short-time glycemic control [2]. Recently, a study stated that TIR is the most paramount indicator for diabetic patients in choosing treatment methods [3]. What is more, other study indicated an association between a high percent of TIR with improvement of hypoglycemic awareness in type one diabetic individuals after intrahepatic islet transplantation [4].

Cardiovascular autonomic neuropathy (CAN) is one of the most shared complications of diabetes, with autonomic imbalance including sympathetic system hyperactivity and parasympathetic system hypoactivity [5]. The ACCORD study confirmed that CAN increased the mortality of type 2 diabetes mellitus (T2DM), the mortality rate of diabetic CAN was 1.55 to 2.14 times than non-CAN, and CAN was related to ascending all-cause and cardiovascular disease mortality [6]. Persistent hyperglycemia and hypoglycemia damage nerves through oxidative stress, eventually leading to the occurrence of autonomic neuropathy [7]. At present, several studies have reported associations of GV metrics assessed by CGM with autonomic neuropathy [8]. What is more, recent researches found an association of TIR with diabetic retinopathy (DR) and diabetic nephropathy (DN) in type 1 diabetes mellitus (T1DM) [9], but the correlation between TIR and CAN has not been studied to date.

The goal of this work was to research whether TIR measured by CGM is connected to the presence and severity of CAN.

## 2. Research Design and Methods

### 2.1. Participants

A total of 349 individuals (age ≥ 18 years) with T2DM admitted to the endocrinology department of the Jinling Hospital, Nanjing University, from April 2018 to August 2019 were recruited, all of whom conformed to the 1999 WHO diagnostic criteria for type 2 diabetes. Evaluating by the standard cardiac autonomic reflex tests (CARTs), there were 228 diabetic individuals without CAN (without confirmed CAN) including absent CAN (*n* = 83 cases) and early CAN (*n* = 145 cases) and 121 diabetic individuals complicated with CAN including definite CAN (*n* = 109 cases) and severe CAN (*n* = 12 cases). The mean age of all individuals was 53.11 ± 12.86 years. Exclusion criteria included (1) patients with acute complications of diabetes, acute stress such as severe infection, trauma and surgery, severe cardiovascular and cerebrovascular diseases, severe respiratory disease, malignant disease, and pregnancy; (2) patients with definite hepatic or gallbladder disease; (3) patients with narcotic and psychotropic drugs, and a recent history of alcoholism. The study was supported by the local ethics committee and all individuals gave informed consent.

### 2.2. Clinical and Biochemical Measurements

General clinical information and physical examination such as age, gender, diabetes duration, history of smoking, hypertension, diabetic retinopathy, and the treatment of diabetes were recorded. Height, weight, systolic blood pressure (SBP), and diastolic blood pressure (DBP) were measured. Body mass index (BMI) was computed. Biochemical measurements such as blood and urine samples were tested after overnight fasting. Hemoglobin A1C (HbA1c), triglyceride (TG), total cholesterol (TC), high-density lipoprotein (HDL), low-density lipoprotein (LDL), fasting blood glucose (FBG), fasting C-peptide, blood urea nitrogen (BUN), and serum creatinine (SCr) were detected.

### 2.3. CGM Parameters

The continuous glucose detection system of MiniMed Company and Meiqi Company was used to monitor glucose for 72 hours continuously, and capillary blood glucose at least four times every day was used to update the monitor according to procedures. Intensive activities were prohibited in the course of glucose monitoring. Based on the original blood glucose data recorded by this system, a number of metrics concerning mean blood glucose (MBG) and glycemic variability (GV), including standard deviation (SD), mean amplitude of glucose excursions (MAGE), coefficient of variation (CV), largest amplitude of plasma glucose excursions (LAGE), average daily risk range (ADDR), and *M* value were tested using the EasyGV Version 9.0R2 published by Oxford University. TIR was assessed as the time percent during a 24-hour period when the glucose is in the range of 3.9-10 mmol/L.

### 2.4. Cardiac Autonomic Function Testing

Standard CARTs were performed on all of the enrolled patients. All operations were performed by the only medical staff. Smoking, drinking, and eating were prohibited and drugs like antihistamines and beta-blockers were not allowed twelve hours before the examination. The CARTs were performed using a standard protocol [10]: [1] Valsalva R-R ratio: determined the maximum R-R interval and the minimum R-R interval after Valsalva maneuver. Valsalva R-R ratio greater than or equal to 1.21 was normal, 1.11~1.20 was borderline, and less than or equal to 1.1 was abnormal. (2) Heart rate (HR) response to deep breathing: measured the maximum and minimum R-R interval during each respiratory cycle and turned into heart rate. Determined the mean of the difference between the maximum heart rate and the minimum heart rate in each of the six deep breathing cycles within 1 minute. The difference value of HR responses to deep breathing greater than or equal to 15 was normal, 11~14 was borderline, and less than or equal to 10 was abnormal. [3] HR response to standing: measured the longest R-R interval in 28~ 32 heart beats and the shortest R-R interval in 13~17 heart beats, converted to heart rate, during patients from lying to standing. The ratio of heart rate between standing and lying position greater than or equal to 1.04 was normal, 1.01~ 1.03 was borderline, and less than or equal to 1.0 was abnormal. [4] Systolic blood pressure response to standing: The difference of systolic blood pressure between lying down and after standing up for 2 minutes was measured. Systolic difference value less than or equal to 10 mmHg (1 mmHg = 0.133 kpa) was normal, 10~20 mmHg was borderline, and greater than or equal to 20 mmHg was abnormal. Each test had a score of 0, 0.5, or, 1 if it was in a condition of normal, borderline, or abnormal range, respectively. Therefore, the minimum and maximum score were 0 and 4, separately. The CAN score 0-0.5 and 1-1.5 was considered absent and early CAN, respectively. The CAN score ≥ 2 with or without orthostatic hypotension was considered severe and definite CAN. In this study, without confirmed CAN included absent and early CAN and with CAN included definite and severe CAN, respectively.

### 2.5. Statistical Analysis

Statistical analysis was applied using the SPSS 22.0 software package. Continuous variables conformed to normal distribution were shown as mean ± standard, while continuous variables with an abnormal distribution were expressed as median (upper and lower quartiles). Categorical data was represented as number (percentage). Student's *t*-test was applied for contrast of two samples with a normal distribution. One-way ANOVA was used for comparison of multiple samples, and Kruskal-Wallis test was used for abnormal distributions. *χ*^2^-test was applied for categorical variables. The Spearman analysis was applied to analyze the relationship between TIR and CART parameters. The binary logistic regression was applied to analyze the independent connection between TIR and CAN (without confirmed CAN vs. CAN) by adjusting age, diabetes duration, sex, blood pressure, lipid situation, SCr, BMI, HbA1c (%), and other GV metrics. Furthermore, the multinomial logistic regression was applied to analyze the independent connection between TIR and different stages of CAN by adjusting age, diabetes duration, sex, blood pressure, lipid situation, SCr, BMI, HbA1c (%), and other GV metrics. *P* < 0.05 was considered statistically significant.

## 3. Results

### 3.1. Clinical Characteristics among Every Stage of CAN Groups

The baseline data of all individuals were exhibited in Table 1. The mean age of all subjects was 53.11 ± 12.86 years, the median (upper and lower quartiles) duration of diabetes was 6 [2, 12] years. According to the CARTs, there were 228 diabetic individuals without CAN (without confirmed CAN) including absent CAN (*n* = 83 cases) and early CAN (*n* = 145 cases) and 121 diabetic individuals complicated with CAN including definite CAN (*n* = 109 cases) and severe CAN (*n* = 12 cases). The prevalence of early CAN, definite CAN, and severe CAN were 41.5%, 31.2%, and 3.4%, respectively. Compared with the absent CAN group, patients with more severe CAN showed increased levels of TG, FBG, SD, MBG, ADDR, and *M* value (*P* < 0.05), and lower levels of TIR (*P* < 0.001). Median (upper and lower quartiles) TIR was 75.15 mmol/L (48.92 mmol/L, 90.49 mmol/L) 72.60 mmol/L (52.18 mmol/L, 84.29 mmol/L), 53.23 mmol/L (33.24 mmol/L, 69.98 mmol/L), and 33.44 mmol/L (1.57 mmol/L, 75.28 mmol/L) in absent CAN early CAN definite CAN, and severe CAN, respectively. In addition, the proportion of diabetic retinopathy was obviously higher with the aggravation of CAN (*P* < 0.05). However, there was no significant difference in HbA1c (%) and the remedies for diabetes among different groups.

### 3.2. The Comparison of Clinical Characteristics by Quartiles (Q1-Q4) of TIR

Further analysis after dividing patients into groups was done according to quartiles of TIR ((Q1) ≤ 41%; (Q2): 41-64%; (Q3): 64-83%; (Q4): >83%). The characteristics were shown in Table 2. Firstly, patients with the highest quartiles of TIR had lower FBG, HbA1c (%), SD, MAGE, MBG, LAGE, ADDR, and *M* value (*P* < 0.001). Of note, for CART parameters, HR variation during position changing, Valsalva maneuver, and deep breathing all increased with ascending quartiles of TIR.

### 3.3. Prevalence of all Stage of CAN in Different Quartiles (Q1-Q4) of TIR

**Figure 1:**
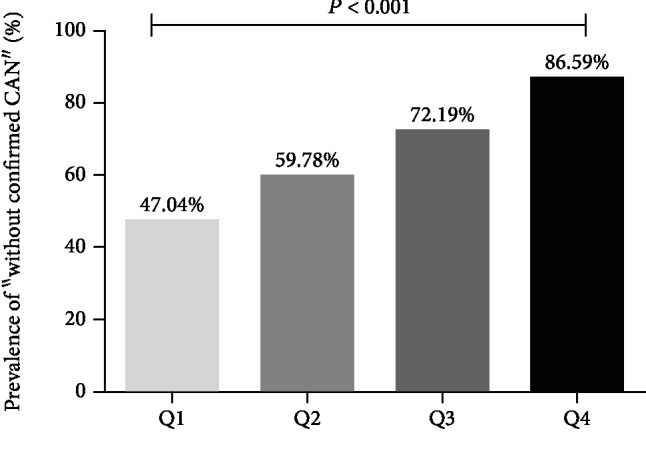
Prevalence of “without confirmed CAN” in different quartiles (Q1-Q4) of TIR. ^a^CAN: cardiovascular autonomic neuropathy. ^b^TIR Q1 ≤ 41%, Q2: 41-64%, Q3: 64-83%, Q4 > 83%. ^c^As shown in this figure, patients were divided into groups according to quartiles of the time in range (TIR), the proportion of “without confirmed CAN” increased with the increase of TIR, (*P* < 0.001). *P* value for the significant difference among the groups was determined by *χ*^2^-test.

**Figure 2:**
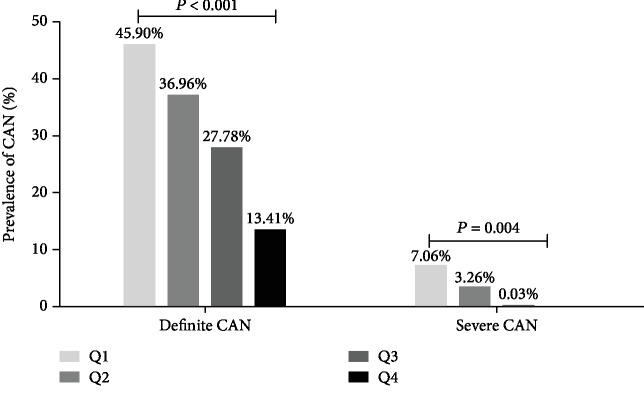
Prevalence of CAN in different quartiles (Q1-Q4) of TIR. ^a^CAN: cardiovascular autonomic neuropathy; ^b^TIR Q1 ≤ 41%, Q2: 41-64%, Q3: 64-83%, Q4 > 83%. ^c^As shown in this figure, patients were divided into groups according to quartiles of the time in range (TIR), the proportion of definite CAN decreased with the increase of TIR, while a similar decreased was found between the prevalence of severe CAN and quartiles of TIR (*P* < 0.05). *P* value for the significant difference among the groups was determined by *χ*^2^-test.

As shown in Figure 1, individuals were classified into groups according to quartiles of the TIR, the proportion of “without confirmed CAN” increased with the increase of TIR (*P* < 0.001) What else, the proportion of definite CAN decreased with the increase of TIR, while a similar decrease was found between the prevalence of severe CAN and quartiles of TIR (*P* < 0.05). (Figure 2).

### 3.4. The Correlation of TIR and Parameters of CARTs

The correlation between TIR and CART parameters were performed with Spearman's analysis. As shown in Table 3, TIR negatively correlated with the total score of CAN (*P* < 0.001) and positively associated with heart rate variation during position changing, Valsalva maneuver, and deep breathing (*P* < 0.05).

### 3.5. Associations between TIR and Various Stages of CAN

Binary logistic regression was used to investigate the independent correlation between TIR and any CAN. After adjusting for age, diabetes duration, sex, blood pressure, lipid profile, SCr, BMI, and HbA1c (%) (model 1), the data revealed that TIR (odds ratio (OR): 0.969, 95% confidence interval (CI): 0.957-0.981, *P* < 0.001) was obviously related to the presence of CAN. The multinomial Logistic regression still found the strong relationship between TIR and manifest or severe stage of CAN (Early CAN: P > 0.05. Manifest CAN: OR: 0.967, 95% CI: 0.952-0.982, P < 0.001. Severe CAN: OR: 0.942, 95% CI: 0.910-0.975, P = 0.001.) Furthermore, the association persisted after adjustment other GV metrics. However, there was no relationship between HbA1c and CAN in all models (Table 4). After categorizing TIR into quartiles, the data showed that the highest quartiles of TIR was associated with the lower presence of CAN compared with the lowest quartiles of TIR after adjusting for age, diabetes duration, sex, blood pressure, lipid profile, SCr, BMI, and HbA1c (%) (model 1) (OR: 0.094, 95% CI: 0.035-0.256, *P* < 0.001, highest vs. lowest). The correlation persisted after adjustment of other GV metrics, but the link between CAN and TIR, as a categorical, was weakened after adjusting MBG (Table 5).

## 4. Discussion

Based on our results, we found a robust association between TIR and CAN independent of HbA1c. What is more, TIR was obviously related to the presence of CAN even after adjusting for GV metrics, including SD and MAGE esc, this result suggested that the value of TIR in predicting the risk of CAN is independent of GV metrics.

Diabetes Control and Complications Trial (DCCT) and the UK Prospective Diabetes Study (UKPDS) [11, 12] has revealed that hemoglobin A1C could be used as the gold standard to assess glycemic control. The DCCT also showed the strong relationship between HbA1c and the danger of long-term diabetic complications. Besides, the primary goal of T2DM management is to decrease glycated HbA1c to 7% or 6.5%. According to the guidelines of the American Diabetes Association, HbA1c is considered an important predictor of chronic diabetes complications [13]. The guidelines also indicated that patients with cardiovascular diseases may have a well-controlled HbA1c. However, HbA1c explained only a portion of the variation in diabetic chronic complications risk. For example, the investigators of the Diabetes Control and Complications Trial (DCCT) found that HbA1c explained only about 11% of the variation in retinopathy risk in the DCCT cohort [14]. At present, most studies demonstrated that HbA1c was a risk factor for the presence and progression of CAN [15]. However, there was no relationship between HbA1c and CAN with adjustment for TIR in our study and there was no difference in HbA1c among different stages of CAN.

Moreover, more and more evidence shows that HbA1c does have certain limitations: Firstly, HbA1c cannot provide information on daily hypoglycemia or hyperglycemia and daily patterns of glycemia. Secondly, the same HbA1c may correspond to a different TIR value [16]. Finally, the accuracy of its measurement is affected by a variety of clinical conditions such as hemoglobinopathies, anemia, uremia, and pregnancy [17].

TIR correlates highly with HbA1c, suggesting that TIR may be used as a novel and promising metric in assessing the risk of diabetes complications and glycemic status in diabetic patients. Compared with HbA1c testing, TIR provides more sensitive and accurate results. As an example, TIR assessment can record acute events of hypoglycemia or hyperglycemia at any time. Obviously, this data cannot be obtained in HbA1c assessment [18]. Omar esc demonstrated that individuals with lower than 80% TIR had worse clinical outcomes than those with higher than 80% TIR, regardless if they had diabetes or not [19]. Recent trials revealed that TIR and HbA1c had similar correlation with chronic diabetic complications. Beck et al. found a strong relationship between TIR and microvascular complications, including diabetic microalbuminuria and retinopathy. The authors also indicated that patients with complications had a decreased TIR (10-12%) in contrast with those who did not. With each 10% decrease in TIR, the risk of retinopathy and microalbuminuria raised by 64% and 40%, separately [9]. Lu et al. explored the relationship between TIR measured by CGM and retinopathy in T2DM. The data showed that individuals with higher TIR had lower risk of developed DR. In addition, TIR had an HbA1c-independent relationship with the prevalence of DR. The association between TIR and the presence of all stages of DR remained unchanged after adjusting GV metrics. This data firstly found the significant effect of TIR on DR independent of GV metrics [20]. A small sample (80 cases) clinical study reported that CAN was negatively associated with percent time in glucose ranging from 70 to 180 mg/dL in type 1 diabetes [21].

A great number of previous studies found the glucose-independent correlation between GV and CAN in newly diagnosed type 2 diabetes. In these study, metrics assessed by CGM such as SD and MAGE esc provided more information beyond HbA1c. Considering that HbA1c had no great difference between CAN and without confirmed CAN group, monitoring glucose patterns more than 24 hours may play a more important role than HbA1c on glucose management in individuals with T2DM and CAN [22–24]. In concert with another study, our results showed that there was no obvious difference in HbA1c (%) among different groups. Compared with absent CAN group, patients with more severe CAN showed increased levels of SD, MBG, ADDR, and *M* value (*P* < 0.05). Logistic regression also revealed TIR was reversely associated with the presence of CAN even after adjusting for GV metrics and HbA1c (%); these results suggested that the value of TIR in predicting the risk of CAN is independent of GV metrics and HbA1c (%).

Short-term hypoglycemia, hyperglycemia, and glycemic fluctuations had an important role in the development of CAN. CAN is the result of the combined action of multiple factors, and its pathogenesis is mainly related to the metabolic disorder, vascular injury, inflammatory reaction, and oxidative stress [25]. The molecular mechanisms related to the association between TIR and CAN have not been clarified yet, which may be the role of oxidative stress. Previous animal and in vitro studies have indicated a robust association between glycemic fluctuations, hypoglycemia, and oxidative stress previously [26], which was also confirmed in several human studies [27].

However, the present study has several limitations. Firstly, considering the small overall sample size in our study, a multicenter larger sample size is needed to confirm the relationship between TIR and CAN. Secondly, we did not conduct the prospective study, making it impossible to investigate the causal relationship between TIR and CAN.

## 5. Conclusion

In conclusion, our work revealed that TIR is associated with the presence of any stages for CAN independent of HbA1c and GV metrics. TIR and other glycemic parameters assessed by CGM have high values and development potential as outcome measures.

## Figures and Tables

**Table 1:** The Clinical characteristics among every stage of CAN groups.

	Absent CAN	Early CAN	Manifest CAN	Severe CAN	*χ* ^2^/*t*/*z*	*P*
*N*	83	145	109	12	—	—
Age (y)	48.28 ± 13.39	52.27 ± 12.45	57.83 ± 10.44	53.92 ± 19.80	9.687	<0.001
Diabetes duration (y)	4.0 (1.0, 10.0)	4.0 (0.8, 10.0)	10.0 (5.0, 16.5)	9.5 (1.3, 18)	31.007	<0.001
Male (*n*, %)	(51, 61.45)	(105, 72.41)	(66, 60.55)	(6, 50.00)	6.110	0.106
Smoking (*n*, %)	(33, 39.76)	(52, 35.86)	(42, 38.53)	(4, 33.33)	0.485	0.922
Retinopathy (*n*, %)	(16, 19.28)	(33, 22.76)	(40, 36.70)	(5, 41.67)	10.360	0.016
SBP (mmHg)	131.58 ± 16.90	129.83 ± 16.06	137.14 ± 18.60	135.00 ± 19.65	3.951	0.009
DBP (mmHg)	82.19 ± 10.85	80.52 ± 9.72	79.86 ± 10.99	76.75 ± 9.96	1.379	0.249
BUN (mmol/L)	5.06 ± 1.31	5.80 ± 2.12	6.67 ± 2.86	5.98 ± 2.51	8.158	<0.001
SCr (*μ*mol/L)	55 (45, 68)	56 (45, 66)	58 (46, 83)	46 (40, 52)	6.244	0.100
TC (mmol/L)	4.65 ± 1.20	4.72 ± 1.23	4.46 ± 1.18	5.09 ± 1.04	1.488	0.218
TG (mmol/L)	1.70 (1.10, 2.64)	1.51 (1.05, 2.40)	1.56 (0.97, 2.02)	2.38 (1.96, 4.13)	8.404	0.038
HDL (mmol/L)	0.99 (0.88, 1.19)	1.04 (0.90, 1.18)	1.04 (0.89, 1.26)	1.01 (0.73, 1.22)	1.558	0.669
LDL (mmol/L)	2.74 (2.07, 3.38)	2.77 (2.26, 3.32)	2.60 (1.89, 3.37)	2.70 (1.71, 3.35)	1.090	0.780
FBG (mmol/L)	7.20 ± 1.90	7.24 ± 2.71	7.82 ± 2.69	11.18 ± 3.64	9.569	<0.001
BMI	24.87 ± 4.00	25.19 ± 3.27	25.15 ± 3.65	26.27 ± 5.75	0.540	0.655
HbA1C (%)	8.67 ± 2.14	9.15 ± 2.39	9.55 ± 2.46	9.88 ± 2.04	2.581	0.053
TIR (3.9-10 mmol/L) (%)	75.15 (48.92, 90.49)	72.60 (52.18, 84.29)	53.23 (33.24, 69.98)	33.44 (1.57, 75.28)	35.487	<0.001
SD (mmol/L)	2.03 (1.50, 2.62)	2.09 (1.70, 2.72)	2.42 (2.02, 3.27)	2.53 (2.08, 2.95)	16.055	0.001
MAGE (mmol/L)	1.08 (0.86, 1.59)	1.10 (0.84, 1.44)	1.19 (0.95, 1.69)	1.08 (0.94, 1.36)	5.457	0.141
MBG (mmol/L)	9.00 ± 1.97	9.34 ± 2.17	10.38 ± 2.36	11.18 ± 2.71	9.323	<0.001
CV	0.24 ± 0.07	0.25 ± 0.08	0.26 ± 0.12	0.24 ± 0.06	1.300	0.274
LAGE (mmol/L)	11.08 (8.77, 13.97)	11.57 (8.97, 15.35)	12.72 (10.48, 17.26)	11.68 (9.90, 16.27)	11.968	0.007
ADDR (mmol/L)	20.19 (11.99, 28.51)	21.41 (15.00, 30.97)	27.58 (20.92, 37.93)	33.60 (19.75, 55.20)	24.836	<0.001
*M* value (mmol/L)	5.18 (2.09, 13.29)	6.54 (3.38, 13.55)	12.23 (6.30, 24.68)	19.58 (6.10, 51.53)	34.801	<0.001
Treatment, (*n*, %)						
No treatment	11 (13.25)	45 (31.03)	25 (22.94)	1 (8.34)	15.207	0.085
OHA	21 (25.30)	38 (26.21)	26 (23.85)	4 (33.33)		
Insulin	27 (32.53)	31 (21.38)	24 (22.02)	3 (25.00)		
OHA & insulin	24 (28.92)	31 (21.38)	34 (31.19)	4 (33.33)		

^a^SBP: systolic blood pressure; DBP: diastolic blood pressure; BUN: blood urea nitrogen; SCr: serum creatinine; TC: total cholesterol; TG: triglyceride; HDL: high-density lipoprotein; LDL: low‐density lipoprotein; FBG: fasting blood glucose; BMI: body mass index; HbA1C: hemoglobin A1C; TIR: time in range; SD: standard deviation; MAGE: mean amplitude of glucose excursions; MBG: mean blood glucose; CV: coefficient of variation; LAGE: largest amplitude of plasma glucose excursions; ADDR: average daily risk range; OHA: oral hypoglycemic agents. ^b^Normally distributed values in the table are presented as the means ± SD, nonnormally distributed values are presented as medians (25% and 75% interquartiles), and categorical variables are presented as frequencies (percentages). ANOVA for comparison of various samples with a normal distribution. Kruskal-Wallis test for abnormal distributions. *χ*^2^ test for categorical variables.

**Table 2:** The comparison of clinical characteristics by quartiles (Q1-Q4) of TIR.

	Quartiles (Q1-Q4) of TIR					
	Q1 (≤41%)	Q2 (41-64%)	Q3 (64-83%)	Q4 (>83%)	*χ* ^2^/*z*值	*P*值
*N*	85	92	90	82		
Age (y)	52.28 ± 13.79	53.89 ± 12.15	53.20 ± 13.03	53.00 ± 12.63	0.232	0.874
Diabetes duration (y)	5.0 (1.0, 12.5)	9.5 (3.0, 12.8)	6.0 (2.0, 15.0)	5.0 (1.0, 10.0)	8.169	0.043
Male (*n*, %)	57 (67.06)	53 (57.61)	60 (66.67)	58 (70.73)	3.661	0.300
Smoking (*n*, %)	33 (38.82)	32 (34.78)	29 (32.22)	37 (45.12)	3.454	0.327
Retinopathy (*n*, %)	30 (35.29)	26 (28.26)	18 (20.00)	20 (24.39)	5.570	0.135
SBP (mmHg)	132.04 ± 19.16	131.98 ± 16.78	132.54 ± 17.01	134.39 ± 16.92	0.351	0.788
DBP (mmHg)	81.22 ± 11.25	79.57 ± 10.15	80.00 ± 10.60	81.37 ± 9.72	0.573	0.633
BUN (mmol/L)	6.18 ± 2.53	5.85 ± 1.82	5.80 ± 2.13	5.78 ± 2.72	0.564	0.639
SCr (*μ*mol/L)	52 (44, 66)	53 (44, 66)	57 (46, 66)	59 (48, 75)	6.471	0.091
TC (mmol/L)	4.64 ± 1.36	4.85 ± 1.25	4.65 ± 1.00	4.37 ± 1.16	2.254	0.082
TG (mmol/L)	1.70 (1.12, 2.32)	1.69 (1.01, 2.46)	1.50 (1.04, 2.27)	1.55 (1.03, 2.23)	0.882	0.830
HDL (mmol/L)	0.98 (0.87, 1.26)	1.06 (0.93, 1.20)	1.05 (0.92, 1.25)	0.99 (0.86, 1.15)	4.676	0.197
LDL (mmol/L)	2.50 (1.90, 3.35)	2.89 (2.28, 3.51)	2.81 (2.29, 3.40)	2.57 (1.89, 3.14)	8.511	0.037
FBG (mmol/L)	9.37 ± 2.80	7.94 ± 3.06	6.87 ± 1.66	5.98 ± 1.51	31.985	<0.001
BMI	24.09 ± 3.93	24.97 ± 3.26	25.91 ± 3.76	25.57 ± 3.48	4.220	0.006
HbA1C (%)	10.97 ± 2.29	9.37 ± 1.87	8.78 ± 2.04	7.58 ± 1.96	39.960	<0.001
SD	2.80 (2.15, 3.92)	2.56 (2.25, 3.07)	2.15 (1.82, 2.51)	1.47 (1.13, 1.78)	147.549	<0.001
MAGE	1.37 (1.09, 1.86)	1.17 (1.01, 1.56)	1.10 (0.91, 1.43)	0.82 (0.63, 1.05)	70.830	<0.001
MBG	12.65 ± 1.82	10.04 ± 1.07	8.52 ± 0.70	7.33 ± 0.84	318.057	<0.001
CV	0.26 ± 0.13	0.27 ± 0.08	0.26 ± 0.07	0.21 ± 0.07	8.122	<0.001
LAGE	15.66 (12.39, 19.23)	13.45 (11.43, 17.05)	11.01 (9.57, 13.47)	8.00 (6.39, 9.50)	138.871	<0.001
ADDR	40.06 (31.40, 52.45)	27.69 (24.32, 32.02)	19.15 (17.04, 24.75)	11.09 (7.82, 14.58)	225.131	<0.001
M value	27.1 3(17.81, 40.90)	11.65 (9.31, 15.80)	5.14 (4.01, 7.02)	1.79 (1.23, 2.86)	277.556	<0.001
SBP response to standing (mmHg)	5 (-6, 11)	4 (-3, 12)	0 (-6, 6)	4 (-4, 9)	5.500	0.139
HR variation during lying to standing	1.08 (1.05, 1.15)	1.11 (1.04, 1.17)	1.11 (1.05, 1.19)	1.13 (1.09, 1.20)	11.525	0.009
HR variation during the Valsalva maneuver	1.10 (1.10, 1.20)	1.10 (1.10, 1.20)	1.20 (1.10, 1.20)	1.20 (1.10, 1.30)	10.103	0.018
HR variation during deep breathing(bpm)	9.00 (6.00, 12.00)	8.00 (6.00, 13.00)	8.50 (5.75, 13.00)	12.00 (6.00, 17.00)	9.094	0.028
Treatment (*n*, %)						
No treatment	16 (18.82)	21 (22.83)	29 (32.22)	16 (19.51)	7.474	0.588
OHA	22 (25.88)	24 (26.09)	19 (21.11)	24 (29.27)		
Insulin	22 (25.88)	24 (26.09)	17 (18.89)	22 (26.83)		
OHA & insulin	25 (29.42)	23 (24.99)	25 (27.78)	20 (24.39)		

^a^TIR: time in range; SBP: systolic blood pressure; DBP: diastolic blood pressure; BUN: blood urea nitrogen; SCr: serum creatinine; TC: total cholesterol; TG: triglyceride; HDL: high-density lipoprotein; LDL: low-density lipoprotein; FBG: fasting blood glucose; BMI: body mass index; HbA1C: hemoglobin A_1_C; SD: standard deviation; MAGE: mean amplitude of glucose excursions; MBG: mean blood glucose; CV: coefficient of variation; LAGE: largest amplitude of plasma glucose excursions; ADDR: average daily risk range; HR: heart rate; OHA: oral hypoglycemic agents. ^b^Normally distributed values in the table are presented as the means ± SD, nonnormally distributed values are presented as medians (25% and 75% interquartiles), and categorical variables are presented as frequencies (percentages). ANOVA for comparison of various samples with a normal distribution. Kruskal-Wallis test for abnormal distributions. *χ*^2^-test for categorical variables.

**Table 3:** The correlation of TIR and parameters of CARTs.

		SBP response to standing (mmHg)	HR variation during lying to standing	HR variation during the Valsalva maneuver	HR variation during deep breathing (bpm)	Total score of CAN
TIR (3.9-10 mmol/L)	*R*	-0.055	0.167	0.139	0.121	-0.261
	*P*	0.302	0.002	0.010	0.024	<0.001

TIR: time in range; CARTs: cardiac autonomic reflex tests; SBP: systolic blood pressure; HR: heart rate.

**Table 4:** Associations between TIR and various stages of CAN.

	Early CAN		Manifest CAN		Severe CAN		Any CAN	
	OR (95% CI)	*P*	OR (95% CI)	*P*	OR (95% CI)	*P*	OR (95% CI)	*P*
Model 1								
TIR	0.995 (0.981-1.010)	0.533	0.967 (0.952-0.982)	<0.001	0.942 (0.910-0.975)	0.001	0.969 (0.957-0.981)	<0.001
HbA1C	*1.116* (*0.960*-*1.296*)	0.153	1.156 (0.972-1.375)	0.102	1.154 (0.782-1.704)	0.471	1.073 (0.938-1.226)	0.303
Model 2								
TIR	0.995 (0.980-1.011)	0.548	0.967 (0.951-0.984)	<0.001	0.940 (0.909-0.973)	<0.001	0.969 (0.957-0.982)	<0.001
HbA1C	1.117 (0.959-1.299)	0.154	1.151 (0.966-1.371)	0.115	1.175 (0.789-1.751)	0.427	1.069 (0.934-1.223)	0.333
SD	0.990 (0.734-1.336)	0.949	1.041 (0.783-1.385)	0.783	0.769 (0.320-1.851)	0.558	1.044 (0.858-1.271)	0.665
Model3								
TIR	0.995 (0.981-1.009)	0.505	0.967 (0.952-0.983)	<0.001	0.941 (0.909-0.974)	0.001	0.969 (0.957-0.981)	<0.001
HbA1C	1.121 (0.963-1.304)	0.140	1.154 (0.969-1.373)	0.108	1.168 (0.787-1.733)	0.441	1.069 (0.935-1.223)	0.330
MAGE	0.928 (0.661-1.304)	0.668	1.048 (0.832-1.320)	0.689	0.784 (0.208-2.953)	0.719	1.082 (0.874-1.339)	0.470
Model4								
TIR	0.996 (0.982-1.010)	0.538	0.967 (0.951-0.982)	<0.001	0.944 (0.912-0.977)	0.001	0.969 (0.957-0.981)	<0.001
HbA1C	1.114 (0.957-1.297)	0.162	1.153 (0.968-1.373)	0.112	1.158 (0.780-1.718)	0.467	1.071 (0.936-1.226)	0.319
CV	1.174 (0.034-40.498)	0.929	1.499 (0.034-66.335)	0.834	0.094 (3.285∗10^−6^-2712.427)	0.652	1.325 0.078-22.473)	0.846
Model 5								
TIR	0.994 (0.979-1.009)	0.421	0.965 (0.949-0.982)	<0.001	0.935 (0.902-0.969)	0.001	0.968 (0.956-0.981)	<0.001
HbA1C	1.124 (0.966-1.308)	0.131	1.160 (0.973-1.381)	0.098	1.204 (0.803-1.807)	0.369	1.077 (0.940-1.233)	0.286
LAGE	0.982 (0.931-1.036)	0.510	0.987 (0.936-1.041)	0.625	0.873 (0.724-1.053)	0.156	0.993 (0.951-1.038)	0.766
Model 6								
TIR	0.994 (0.975-1.013)	0.523	0.961 (0.941-0.983)	<0.001	0.935 (0.894-0.978)	0.003	0.965 (0.948-0.981)	<0.001
HbA1C	1.119 (0.961-1.303)	0.149	1.169 (0.981-1.394)	0.081	1.169 (0.788-1.734)	0.438	1.083 (0.945-1.240)	0.250
ADDR	0.996 (0.963-1.029)	0.789	0.986 (0.950-1.023)	0.448	0.982 (0.911-1.058)	0.636	0.989 (0.961-1.018)	0.460
Model 7								
TIR	0.999 (0.978-1.019)	0.896	0.973 (0.951-0.995)	0.015	0.943 (0.903-0.985)	0.008	0.972 (0.959-0.986)	<0.001
HbA1C	1.114 (0.959-1.295)	0.158	1.154 (0.970-1.374)	0.105	1.147 (0.776-1.694)	0.492	1.073 (0.938-1.227)	0.305
*M* value	1.008 (0.969-1.050)	0.683	1.014 (0.975-1.056)	0.481	1.004 (0.949-1.063)	0.884	1.007 (0.993-1.021)	0.333

^a^TIR: time in range; HbA1C: hemoglobin A1C; SD: standard deviation; MAGE: mean amplitude of glucose excursions; CV: coefficient of variation; LAGE: largest amplitude of plasma glucose excursions; ADDR: average daily risk range; HR: heart rate; CI: confidence interval. ^b^Model 1 was adjusted for age, diabetes duration, sex, blood pressure, lipid profile, SCr, BMI, and HbA1c (%); model 2 was adjusted for variables as in model 1 and for SD; model 3 was adjusted for variables as in model 1 and for MAGE; model 4 was adjusted for variables as in model 1 and for CV; model 5 was adjusted for variables as in model 1 and for LAGE; model 6 was adjusted for variables as in model 1 and for ADDR; model 7 was adjusted for variables as in model 1 and for *M* value.

**Table 5:** Association between quartiles of TIR and CAN.

	*β*	S.E.	Wald	*P*	OR (95% CI)
Model 1					
TIR Q1	—	—	—	—	1 (reference)
TIR Q2	-0.686	0.367	3.483	0.062	0.504 (0.245-1.035)
TIR Q3	-1.265	0.393	10.347	0.001	0.282 (0.131-0.610)
TIR Q4	-2.364	0.511	21.394	<0.001	0.094 (0.035-0.256)
Model 2					
TIR Q1	—	—	—	—	1 (reference)
TIR Q2	-0.676	0.371	3.332	0.068	0.508 (0.246-1.051)
TIR Q3	-1.250	0.403	9.637	0.002	0.287 (0.130-0.631)
TIR Q4	-2.337	0.532	19.307	<0.001	0.097 (0.034-0.274)
SD	0.018	0.099	0.033	0.856	1.018 (0.838-1.237)
Model 3					
TIR Q1	—	—	—	—	1 (reference)
TIR Q2	-0.649	0.370	3.084	0.079	0.523 (0.253-1.078)
TIR Q3	-1.229	0.395	9.663	0.002	0.292 (0.135-0.635)
TIR Q4	-2.312	0.514	20.241	<0.001	0.099 (0.036-0.271)
MAGE	0.071	0.110	0.419	0.517	1.074 (0.866-1.331)
Model 4					
TIR Q1	—	—	—	—	1 (reference)
TIR Q2	-0.675	0.369	3.357	0.067	0.509 (0.247-1.048)
TIR Q3	-1.254	0.394	10.120	0.001	0.285 (0.132-0.618)
TIR Q4	-2.387	0.514	21.538	<0.001	0.092 (0.034-0.252)
CV	-0.646	1.471	0.193	0.661	0.524 (0.029-9.372)
Model 5					
TIR Q1	—	—	—	—	1 (reference)
TIR Q2	-0.701	0.370	3.598	0.058	0.496 (0.240-1.024)
TIR Q3	-1.303	0.405	10.358	0.001	0.272 (0.123-0.601)
TIR Q4	-2.425	0.534	20.615	<0.001	0.088 (0.031-0.252)
LAGE	-0.009	0.022	0.162	0.688	0.991 (0.948-1.036)
Model 6					
TIR Q1	—	—	—	—	1 (reference)
TIR Q2	-0.720	0.404	3.169	0.075	0.487 (0.221-1.075)
TIR Q3	-1.319	0.474	7.750	0.005	0.267 (0.106-0.677)
TIR Q4	-2.439	0.631	14.916	<0.001	0.087 (0.025-0.301)
ADDR	-0.003	0.014	0.041	0.839	0.997 (0.971-1.025)
Model 7					
TIR Q1	—	—	—	—	1 (reference)
TIR Q2	-0.417	0.426	0.957	0.328	0.659 (0.286-1.519)
TIR Q3	-0.917	0.487	3.541	0.060	0.400 (0.154-1.039)
TIR Q4	-1.958	0.602	10.583	0.001	0.141 (0.043-0.459)
*M* value	0.015	0.013	1.306	0.253	1.015 (0.990-1.040)

^a^TIR: time in range; SD: standard deviation; MAGE: mean amplitude of glucose excursions; CV: coefficient of variation; LAGE: largest amplitude of plasma glucose excursions; ADDR: average daily risk range; HR: heart rate; CI: confidence interval. ^b^Model 1 was adjusted for age, diabetes duration, sex, blood pressure, lipid profile, SCr, BMI, and HbA1c (%); model 2 was adjusted for variables as in model 1 and for SD; model 3 was adjusted for variables as in model 1 and for MAGE; model 4 was adjusted for variables as in model 1 and for CV; model 5 was adjusted for variables as in model 1 and for LAGE; model 6 was adjusted for variables as in model 1 and for ADDR; model 7 was adjusted for variables as in model 1 and for *M* value. ^c^TIR *Q*1 ≤ 41%, Q2: 41-64%, Q3: 64-83%,*Q*4 > 83%.

## Data Availability

The datasets generated during and/or analyzed during the current study are available from the corresponding authors and the senior author on reasonable request.
